# Update of Immunosenescence in Cerebral Small Vessel Disease

**DOI:** 10.3389/fimmu.2020.585655

**Published:** 2020-11-18

**Authors:** Banghao Jian, Mengyan Hu, Wei Cai, Bingjun Zhang, Zhengqi Lu

**Affiliations:** ^1^ Department of Neurology, Center for Mental and Neurological Disorders and Diseases, The Third Affiliated Hospital of Sun Yat-sen University, Guangzhou, China; ^2^ Center of Clinical Immunology, Center for Mental and Neurological Disorders and Diseases, The Third Affiliated Hospital of Sun Yat-sen University, Guangzhou, China

**Keywords:** aging, immunosenescence, cerebral small vessel disease, pathogenesis, inflammaging

## Abstract

Aging of the central nervous system (CNS) is closely associated with chronic sterile low-grade inflammation in older organisms and related immune response. As an amplifier for neuro-inflammaging, immunosenescence remodels and deteriorates immune systems gradually with the passage of time, and finally contributes to severe outcomes like stroke, dementia and neurodegeneration in elderly adults. Cerebral small vessel disease (CSVD), one of the major causes of vascular dementia, has an intensive connection with the inflammatory response and immunosenescence plays a crucial role in the pathology of this disorder. In this review, we discuss the impact of immunosenescence on the development of CSVD and its underlying mechanism. Furthermore, the clinical practice significance of immunosenescence management and the diagnosis and treatment of CSVD will be also discussed.

## Introduction

Cerebral small vessel disease (CSVD), a considerable health care problem, contains a wide spectrum of cerebrovascular diseases that primarily affect capillaries, small arteries and small veins in the brain, and brings a serious hazard to aging societies (1). Arteriolosclerosis is the most popular and extensive subtype of CSVD and closely related to the overall health status of the body like aging and hypertension. Thus, such type is also called age-related and vascular risk-factor-related small vessel disease (2). Mounting evidence indicated that arteriolosclerosis is the major cause of ischemic stroke, intracerebral hemorrhage, dementia and raises mortality in elderly people, and the term CSVD in this review is majorly used to discuss arteriolosclerosis (3). The neurobiological basis of the underlying mechanism of CSVD is poorly understood now. However, chronic inflammation, induced by either immune cells or non-immune cells, is closely associated with the aging process of cerebral vessels and related brain metabolism and draws a substantial amount of attention. Therefore, it is of great potential and significance to investigate the role of immune response during aging in the development of CSVD and corresponding cerebral injury.

To better understand the immune system alteration during aging, the term “immunosenescence” raises and represents the deterioration of multiple immune cells and function change of key molecules like cytokines, chemokines and extracellular matrix components (4). It is renowned that immunity acts an emerging role in the pathological process of CSVD in elderly (5). The peripheral immune system, involving lymphocytes, cytokines and antibodies, contributes to the vascular disorganization, blood brain barrier (BBB) leakage and immune cells infiltration. Importantly, damaged cells suffer a special stage called senescence-associated secretory phenotype (SASP) from normal states to irreversible aging states. SASP could be found in infiltrated immune cells and residential cells, including endothelial cells, pericytes, neurons and glial cells during CSVD. All the components could form a complex regulatory network and play a crucial role in the pathophysiology of CSVD (6, 7). Actually, immunosenescence has a bidirectional impact on multiple disorders development, which often refers to immunosuppression or immune activation. Such pathological process tends to activate an immune response in CSVD and contribute to chronic low-grade inflammation in the CNS, named neuro-inflammaging.

Performing in-depth exploration of the cellular and molecular level of immunosenescence could help physicians and specialists to make a clinical decision and better predict the prognosis of CSVD patients. Through screening key molecules in pathways related to immunosenescence, a regulatory network and vital point target genes could be constructed and found. Specialists could focus on these key modules to build a prognostic model for CSVD patients and provide more precise clinical plan. In addition, drug development could be more time-saving and lower economic toxicity based on these fundamental researches. However, the contribution of immunosenescence in the initiation and progression of CSVD remains obscure and an unmet need for further exploration. This review focuses on the impact of immunosenescence on the development of CSVD and its underlying mechanism, and the meaning of immunosenescence management for CSVD in clinical practice is also discussed.

## Immunosenescence is a Promising Amplifier for Neuro-Inflammaging

The concept of immunosenescence states the aging and functional decline throughout the whole immune system. It usually accompanies with a chronic low-grade inflammation status termed inflammaging whose pro-inflammatory mediates remarkably increase (8). This process not only involves the immune system, but tissues such as senescent endothelium, pericytes and adipose cells also. The immune system is composed of innate and adaptive parts, both of which dramatically change in the aging process (9).

### Immune System Alterations in Aging Process

Both adaptive immunity and innate immunity undergo significant changes during the natural process of aging and consequently cause a series of physiological and molecular changes (**Table 1**). The cell transformation of lymphocytes, along with the antibody and cytokines secretion, represents the function of adaptive immunity while diminished function and phenotype shift towards proinflammatory subtype of macrophages mainly consist of the innate immunity alteration.

**Table 1:** Summary of immune changes in immunosenescence.

Immunity	Cell	Changes	Study
Adaptive	T cell	- TCR pool diminishes	Kilpatrick et al. (10)
		- Subtype changes	Li et al. (11) Jagger et al. (12) Raynor et al. (13)
		- Inflammatory suppression weaken	Fessler et al. (14) Thomas et al. (15)
		- Pro-inflammatory mediates production increase	Hu et al. (16) Singh & Newman (17)
	B cell	- Subtype changes	Bulati et al. (18) Palma et al. (19)
		- Pro-inflammatory mediates production increase	Frasca & Blomberg (20)
Innate	Monocyte/macrophage	- Chemotaxis/phagocytosis decrease	Antonaci et al. (21) Mahbub et al. (22)
		- Subtype changes	Seidler et al. (23) Sadeghi et al. (24)
		- Pro-inflammatory mediates production increase	Sadeghi et al. (24) Olivieri et al. (25) Jacinto et al. (26)
	Dendritic cell	- Number decreases	Della et al. (27)
		- Pro-inflammatory mediates production increase	Splunter et al. (28)
	Microglia	- Subtype changes	Yao & Zhao (29)
		- Pro-inflammatory mediates production increase	Mecca et al. (30) Vida et al. (31)
	Neutrophils	- Chemotaxis/phagocytosis decrease	Niwa et al. (32)
	NK cell	- Number increase	Gounder et al. (33)
		- Pro-inflammatory mediates production increase	Camous et al. (34)

The adaptive immune system contains cellular immunity and humoral immunity, composed of T cells and B cells in different subpopulations. According to the initial of lymphocytes, T cells origin from bone marrow, and then differentiate and mature in thymus. Later, these functional cells release into circulation and migrate to peripheral immune organs and tissues. Commonly, T cells can be subdivided into 4 subgroups based on their distinct function: helper T cell, cytotoxic T cell, regulatory T cell (Treg) and memory T cell. These subtypes participate in pathogen identification, cytokines secretion, immune memory, pathogen killing and immunoregulation, whose function was impaired at varying levels in immunosenescence process. Meanwhile, the cell counts of specific subtypes in functional immune organs including thymic and ratio of different T cell types, which is widely found, will also change in elderly. For example, the CD4 naïve T cells continuous decline with the thymic involution while the number of T cells still remains steady at the periphery *via* compensatory proliferation, resulting in the loss of diversity of T cell receptor (TCR) pool (10, 35). Besides, both the increase of the CD4/CD8 ratio and CD8^+^CD28^-^ T cells was observed in elderly people (11, 36).

Great attention has been paid to uncover the underlying roles of some subtypes of T cells as more accurate and precise detecting method develops and clear definition has been made to these cell types. According to the recent studies, the T cell types that only account for a small part the total population may also make great sense and have tight connection with other cells or stromal components which could be also called microenvironment. It should be noted that the investigation on Tregs, a newly research hotspot in T lymphocytes, is still disputing. Some studies illustrate the downward number of both natural Tregs and induced Tregs in the aging process, while others found that the population of CD4^+^ Treg increased remarkably in immunosenescence (12, 13). Genetic and epigenetic modification also weakens the capacity of T cells (37). In all, complex cellular biological changes make the host susceptible to infection (37) and new technologies like single cell sequencing would discover a virgin land in this area and help researchers focus on the underlying mechanisms.

Senescent T cells could participate in inflammaging process as a double agent role, which is based on its immunosuppression function in the early stage and active function in late stage (38). First, loss of TCR function affects the contact of myeloid-derived suppressor cell (MDSC) responsible for inflammatory suppression (38, 39). In late stage, inflammatory suppression was impaired owing to the increase of CD8^+^CD28^-^ Tregs because of their weaker inflammatory resolution and shorter lifespan compared with CD8^+^CD28^+^ Tregs (14). Thymic atrophy impairs the negative selection, contributing to increasing self-antigen-recognizing conventional T cells (15). The imbalance of T cell subtypes causes the disorder of inflammatory factor production such as interferon-inducible protein-10, interleukin (IL)-6 and IL-8, increasing with age and exacerbate the inflammaging state (16, 17). The DNA damage resulting from telomere attrition in senescent T cell activates the NF-κB pathway, contributing to consistent uplifting of pro-inflammatory mediators (38).

Similarly with T cells, the aging changes of B cells include the reduction of naïve B cells together with expansion of memory B cells (18, 19). Senescent B cells with altered B cell receptors and functions are associated with higher cytokines production and antibody presentation (40). Besides, B cells under chronic inflammatory simulation tend to produce proinflammatory cytokine and pathogenic antibodies (20).

The innate immune system consists of several physical barriers and various cells, including monocytes/macrophages, neutrophils, eosinophils, basophils, dendritic cells and innate-like lymphocytes like NK cells (41). Although less attention has been paid, transformation of innate immunity is earlier and stronger than adaptive immunity during the senescent process, indicating its crucial role in the natural aging and pathology.

Monocytes, macrophages, dendritic cells (DCs) and microglia are responsible to chemotaxis, phagocytosis, secretion and antigen presentation, but these functions decline in elderly individuals. Those over 55 turned out to have more peripheral monocytes with impaired chemotaxis and phagocytosis. Moreover, the inflammatory pathway and immune response of macrophages are downregulated and suppressed (21, 22). The number of myeloid DCs and monocyte precursors progressively decreases with increasing age, though the number of classical CD14^+^CD16^-^ monocytes remains stable (27). A class of CD14^+^CD16^+^ monocyte subset, with downregulated expression of HLA-DR and CX3CR1, increases with age significantly (23, 24). Interestingly, some shifts result in the weaker defense ability, while other changes result in the permanent low-level inflammatory environment. The alteration of subtypes and upregulation of pro-inflammatory cytokine expression is present in both aging DCs and microglia, aggravating the inflammation in CNS (28–31). Besides, dysfunction of microRNA results in higher production of inflammatory cytokines such as IL-6, and reactive oxygen species (ROS), noted in both steady and active state of monocytes during aging (24–26).

Neutrophils, as an essential part of innate immunity performing cytotoxic effects and phagocytosis, are closely related to several inflammatory diseases. The function and lifespan of neutrophils are regulated by various cytokines: IL-2 prolongs the lifespan and promotes inflammation while tumor necrosis factor (TNF)-α induces the apoptosis. Senescent neutrophils show decreased phagocytic, chemotactic ability and ROS production while the adherent ability appears to remain still (38, 42). Although these changes result in down-regulation of the inflammatory response, the increased neutrophil generation enhances the inflammaging (43). Overexpression of PI3K results in the inaccurate migration of neutrophils and damages of normal tissues (43, 44). Besides, elevated level of IL-6 and IL-8 enhances neutrophils activation which would conversely affect inflammation outcome (45).

NK cells are differentiated from myeloid-lymphoid stem cells, and they perform unspecific killing function targeting abnormal cells like tumor cells, injured cells, and virus-infected cells *via* multiple cytotoxic effects. Commonly, senescent NK cells have weaker proliferative ability and larger cytotoxic subpopulation (33). When immune response initials, cytotoxicity of NK cells from elderly donors are weaker, which results from the alteration of receptors or production of enzymes (46). Further, NK cells were overactive in aging process *via* alteration of TLR function and shift of subtypes, and they act as effector in inflammaging (34).

### Immunosenescence Plays an Important Role in Age-related CNS Disease

**Figure 1:**
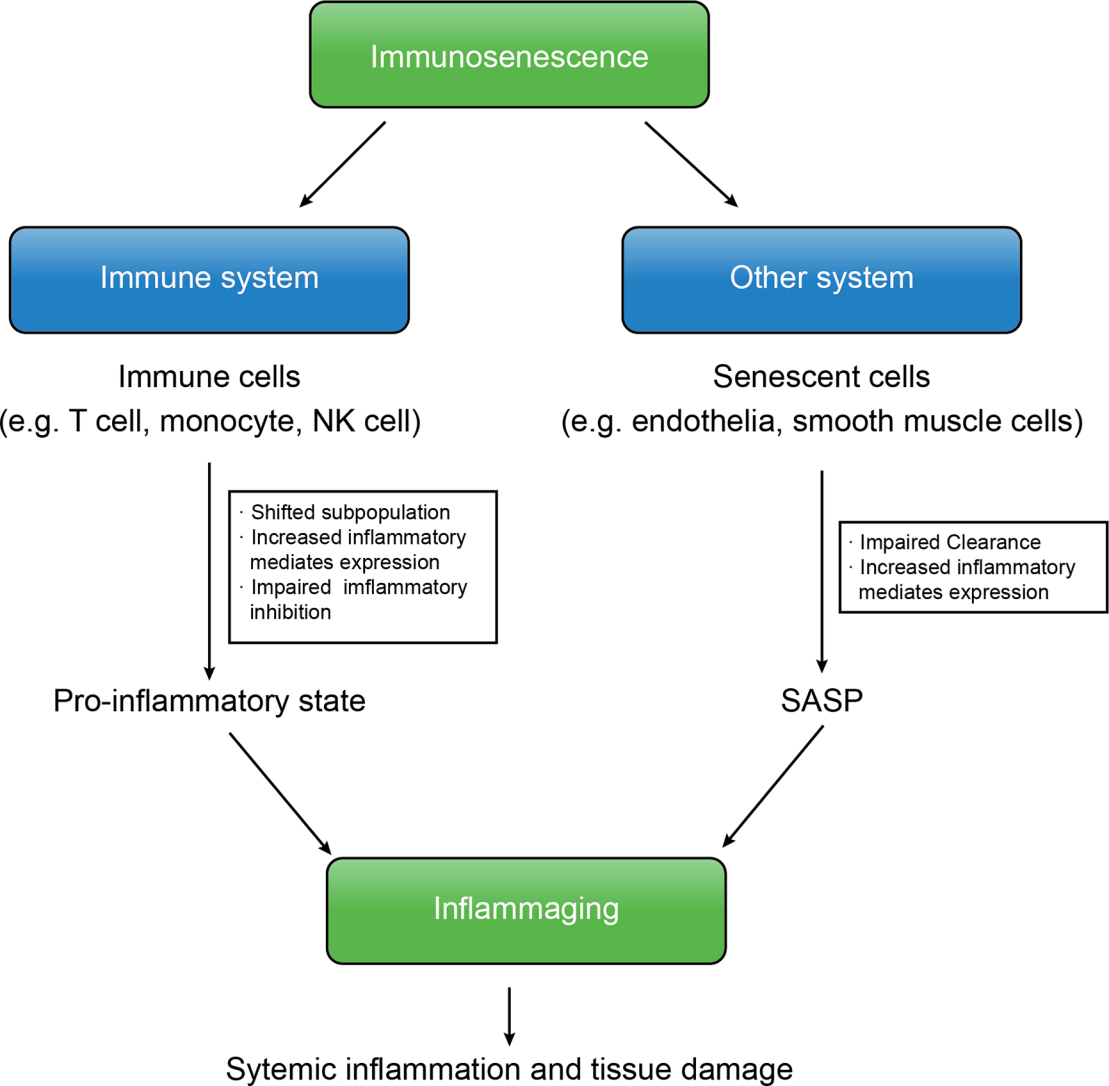
The immunosenescence and inflammaging are interconnected. In immunosenescence, accumulation of senescent cells produce SASP, increased expression of inflammatory mediates of both immune cells and senescent cells and the insufficient downregulation of inflammation shift the body into the inflammaging state that results in further systemic inflammation and tissue damage, leading to several aged-related diseases.

Immunosenescence is the core of various aged-related CNS diseases, including cerebral vessel diseases and degenerative neurological diseases. At the beginning of immunosenescence, senescent cells fail to be cleared, resulting in accumulation of abnormal cells and cell fragment and induction of SASP (47). On this basis, the systemic pro-inflammatory state damages brain tissue, which is worse owing to the pro-inflammatory mediators produced from SASP (**Figure 1**).

Alzheimer’s disease (AD), the pathogenesis of which remains unknown, has a great connection with age. Most scientists believe in beta amyloid hypothesis that defines the deposition of β amyloid peptide (Aβ) as initiator, interrupting the cellular metabolism and eventually progressing into AD. Immunosenescence is also considered as a candidate mechanism of AD, because the accumulation of Aβ is closely associated with impaired clearance ability and cytotoxic effect of senescent microglia (48). Aβ deposition interacts with glial cells, pericytes, and neurons, modifies BBB and leads to immune cell infiltration (49). Those infiltrated cells and inflammatory mediators induce a proinflammatory environment around the lesion, which eventually leads to neuroinflammation and neurodegeneration (50, 51). Current study concluded that the systematic immune response also involves in the neuroinflammation for which there is a large amount of evidence suggesting a proinflammatory state in AD patient, which implicates the complicated influence of immunosenescence in this disease (50).

Parkinson’s disease (PD) is another common neurodegenerative disease in elderly that results from the degeneration of dopaminergic (DA) neurons in substantia nigra, leading to severe motor disorders. Mounting evidence suggested that oxidative stress and inflammation injure the DA neurons, in which microglia and astrocytes also play important roles (52–54). Early study suggests that humoral immunity targets DA neurons which promotes the neuroinflammation and neurodegeneration in PD patient, agreeing with the activated microglia and elevated cytokines observed in neurotoxin-based PD model (55, 56). The therapy that focuses on balance between pro- and anti-inflammation provides a promising therapeutic strategy of PD (57). Pharmacological inhibition of oxidation and inflammation reverses the function of monoaminergic synthesis, which also supports this idea (58).

Cerebral vascular disease is a class of disease involving brain vessels, including vasculitis, cerebral amyloid angiopathy, subacute arteriosclerotic encephalopathy and CSVD, some of which have been prone to be associated with immunosenescence and inflammaging (59, 60). There lies a gap in CSVD, which we will discuss in the next section.

## Senescence of Immune Cells, Non-Immune Cells and BBB Dysfunction in Age-Related CSVD

**Figure 2:**
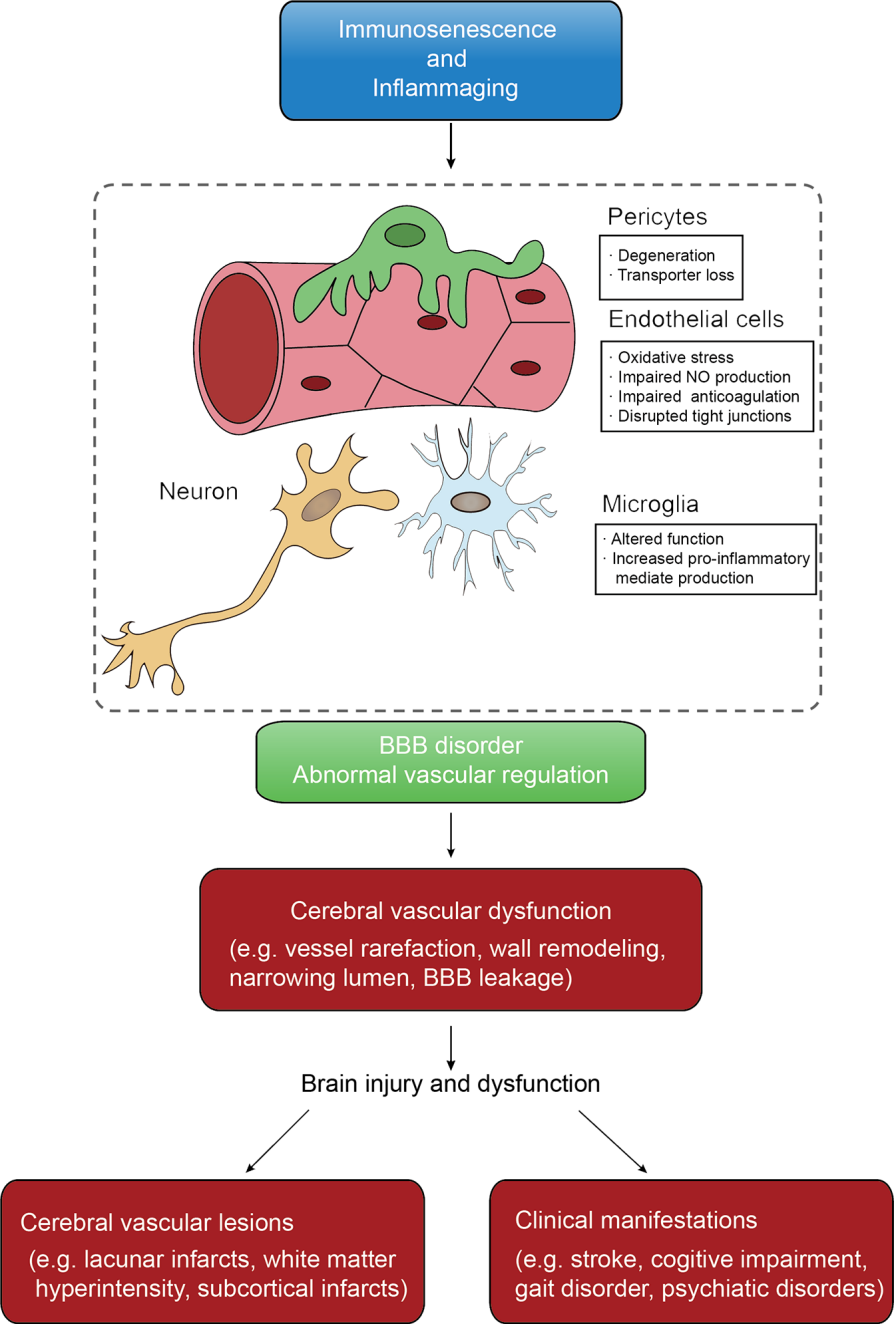
The conceptual model of immunosenescence and inflammaging mechanism of CSVD. Immunosenescence and inflammaging affect both vascular function and BBB intactness that are keys of CSVD initiation. Cerebral vascular dysfunctions are various that further lead to pathological changes that can be found on neuroimaging and clinical features.

With ever-increasing life expectancy worldwide, the number of individuals living in the community with age-related diseases, especially CSVD (ArCSVD), will increase (61). The main manifestations of ArCSVD include stroke, cognitive declines, gait disorder, psychiatric disorders and urinary incontinence, and its sequelae would impose a considerable burden on families and society. ArCSVD is significantly associated with risk factors like aging, arterial hypertension, smoking, diabetes mellitus, obstructive sleep apnea (62–64). Besides, ArCSVD mainly affects the small blood vessel of the cortex or medulla, including small arteries, arterioles, capillaries, venules and small veins. The initial pathological characteristics are endothelial proliferation, small vessel wall thicken and arterial disorganization, and then develop into arteriolosclerosis and lipohyalinosis, which underline the pathological basis of ArCSVD. From an imaging perspective, ArCSVD is characterized by features like lacunar infarcts, white matter hyperintensity, subcortical infarcts, cerebral microbleedings, perivascular spaces, intracerebral hemorrhage and cerebral atrophy (2, 65, 66). Unfortunately, pathological mechanisms of ArCSVD are incompletely understood yet. According to recent studies, immunosenescence roles in endothelial dysfunction and blood-brain barrier disorder earn sustainable attention, which seems to be the possible candidate for further study (67, 68) (**Figure 2**).

### Senescence of Immune Cells

Immune cells senescence in the early stage of ArCSVD intrinsically suppresses normal immune response and as a result increases infection risk, whereas the subsequent SASP would produce proinflammatory cytokines, including IL-1β, IL-6, and IL-8, and consequently accelerate and aggravate endothelial injury and BBB leakage. Each subsets of immune cells display specific function of immunosenescence in ArCSVD.

As one important type of phagocytes, microglia take responsibility for removing injured or dead neurons, glial cells and debris of the myelin sheath in CNS. Meanwhile such cells also act as a stabilizer of CNS structure and maintain BBB integrity and promote injured tissue recovery (69). However, accumulating evidence confirmed that microglia would become senescent and dysfunction, most likely contributing to low-grade inflammation in CNS and aged-related neurodegenerative diseases (70, 71). From the perspective mechanisms, hypofunction of TREM2-DAP12 and CX3CL1-CX3CR1 axes plays a crucial role in the loss of phagocytosis and inflammatory modulation (30, 31). Senescent microglia suffers from the loss of responsiveness, migration and phagocytosis, leaving the accumulation of senescent cells and debris as a source of chronic inflammation that damage cerebrovascular structures and neurons (71, 72). Up-regulation of age-dependent inflammatory pathways are related to pro-inflammatory shift and increasing cytokine production of microglia (73, 74). The cross-talk among astrocytes, neutrophils, monocytes and macrophages, exacerbating the immune cell infiltration and inflammatory response consequently (75). In conclusion, senescent microglia activate inflammaging by tilting toward pro-inflammatory state, which is responsible for neuron degeneration and BBB leakage (76).

In case of infiltrated immune cells augmented in the early onset ArCSVD, including macrophages, neutrophils, T cells and NK cells, are responsible for the inflammation, endothelial dysfunction and ischemia of the area (77). As we mention above, the infiltration of senescent immune cells with increasing inflammatory mediates expression and altered function would aggravate the inflammation storm in regions of the lesion. ROS and other cytotoxic products from infiltrating immune cells enhance the oxidative stress of endothelial cells, resulting in further vessel tone dysfunction and vascular remodeling (78). While localized inflammation is a crucial damage to the brain, it is also essential that the peripheral immune response could aggravate the inflammation. According to a community-based study, systemic inflammation is related to white matter microstructural integrity among older adults (79). Furthermore, anti-endothelial antibodies were found in ArCSVD patients, implying the relationship between B cell activation and endothelial dysfunction (80).

Taken together, the senescence of immune cells have a strong connection with dysfunction of glia cells and endothelial cells. Non-immune cells under the damage of inflammatory environment finally become dysfunctional and promote the progress and development of ArCSVD.

### Senescence of Non-Immune Cells

Senescent residential non-immune cells in CNS, including endothelial cells, astrocyte, pericytes, and oligodendrocytes, are also found to play crucial roles in destruction and dysfunction of BBB in ArCSVD. Moreover, these senescent cells can be recognized by immune cells and activate a further response.

Endothelium is an important system regulating vessel remodeling, vascular tone, balance of inflammatory and coagulation. Of note, it is a fact that endothelial dysfunction is attributed to be a key mechanism in ArCSVD (81). Apart from the increasing age, there is cogent evidence supporting that two interconnected mechanisms—cellular oxidative stress and low-grade inflammation — also contribute to endothelial senescence (82).

Endothelial dysfunction mainly reflects in vessel tone changes. Multiple factors are known as the contributor in vessel tone regulation, but eNOS-derived NO is usually considered to be the most important mechanism. NO plays an important role in relaxation of vascular smooth muscle cells and preservation of cerebral blood flow (82). The inhibitory effect of NO restrains platelets from aggregation and adhesion, and the release of platelet-derived growth factor that stimulates smooth muscle cell proliferation (83). Meanwhile, NO also prevents relating immune cells from activation of NF-κB and formation of inflammatory factors (84, 85). However, the changes of eNOS signaling on transcriptional and post-transcriptional level resulting from hypertension, angiotensin II and aging, suppress the function of eNOS by reducing NO synthesis (86–88). The loss of NO synthesis results in vessel tone disorder, reduces the cerebral blood flow, exacerbates oxidative stress and vulnerability of acute ischemia. Furthermore, there is a vicious cycle in the senescent endothelium (89). Activating TLR-NF-κB pathway results in subsequently cytokines production including IL-1α, IL-1β, IL-6, IL-8, IL-10, IL-12, TNF-α, and IFN-γ (25, 90, 91). Pro-inflammatory cytokines as well as the NF-κB protein induces the expression of NADPH oxidase, contributing to the increase production of ROS (92). These oxidative stress productions act as a positive feedback that increases NF-κB activity, meanwhile they also activate circulating and residential immune cells (89). Free radicals would impair NO production by causing eNOS uncoupling directly, the amplifying oxidative stress further worsening the dysregulation of NO production (93). Taken together, it’s trapping in a vicious cycle of oxidative stress and inflammation that progressively damage the endothelial function which consequently develops into cerebrovascular dysfunction.

Pathologically, senescent epithelium results in impairment of proliferation and angiogenesis, leading to abnormal vessel remodelling (94). Vessel rarefaction is commonly observed in hypertension and ArCSVD patient, suggesting the reduction of the blood vessel in tissues (95). Decreased density and length of capillary are found in the cerebral cortex of aging people. Moreover, morpho-functional changes of capillary bed of the cortex, reduction of external diameter, and increased wall-to-lumen ratio and resistance are found in hypertensive patients (96, 97). The consequences of cerebrovascular microstructure remodeling are ischemic changes in the regions supplied by the responsible blood vessels, showing as lacunar infarcts, white matter hyperintensity or perivascular space in imageology.

Pericytes are isolated contractile cells that regulate cerebral blood flow and maintain BBB (98). Pericytes participate in the formation of capillary basement membrane, while multiple signaling pathways between pericytes and astrocytes also exert an essential effect on BBB integrity (99, 100). Dysfunction of pericytes contributes to the aged-related cerebrovascular diseases. According to a current study, the loss of pericyte coverage is reported in aged mice, which may result from hypertensive induced oxidative stress (101). While another study found that the loss of pericyte in aging mice and attributed it to the glutamine pathway and ischemia as pericyte is sensitive to ischemia (102). Moreover, pericytes are also under the effect of inflammation. For example, some investigators observed increasing permeability of BBB resulting from IL-1β-induced pericytes, and come to the conclusion that dysfunction of pericytes and inflammation may damage the integrity of BBB (103). What’s more, loss of pericyte coverage reduces microcirculation, breaks down BBB, exacerbates oxidative stress and cause neurodegeneration, which is observed in an experiment of pericyte-deficient mice (99). Thus, pericyte dysfunction is a crucial part should not be neglected in ArCSVD.

### Dysfunction of BBB

It is obvious that BBB disorder, on the basis of endothelial dysfunction and pericyte disorder, plays a critical role in ArCSVD. BBB is composed of basic membrane, astrocytes end feet, pericytes and endothelial cells with tight junction (68). The incidence of BBB leakage increases with risk factors like toxicity, trauma, age and hypertension, and such injury is mainly related to cell response induced by immunosenescence (104, 105). Although the pathophysiology of BBB is complicated and intricate, the major process nevertheless is recognized, and the most identical initiation is endothelial dysfunction whose commonest outcome is BBB.

In the context of cerebrovascular dysregulation, chronic exposition of high shear force leads to the alteration of the tight junction and increased permeability of BBB (106). As a result, immune cells and plasma components could enter the brain parenchyma and trigger sterile inflammation (107). Subsequently, oxidative stress and inflammatory cytokines induced by the immune cells aggravate the local inflammatory response and hinder the damage repair (85, 108). Furthermore, damage of other glial cells and its production of large quantities of inflammatory mediators and toxic substances could destroy the barrier system (109).

Accordingly, a broad range of pathophysiological changes in BBB disorder would finally contribute to white matter lesions and other secondary damage in CSVD while imaging shows a phenomenon termed as white matter hyperintensities (WMH) in the clinical context (110). The degree of BBB leakage is commonly assessed according to the signal of WMH – the greater the range of WMH, the more severe is the BBB leakage (111).

## Candidate Markers of Immunosenescence in ArCSVD for Clinical Diagnosis

The clinical performance of ArCSVD is complicated and variable, and diagnosis of such disease relies on the imaging findings. However, imaging exists an inherent drawback that only the late stage patient whose injury is mostly irrecoverable could perform remarkable imaging manifestations, indicating an unmet need for developing one or more neo-markers for screening patients at early stage. Thereafter, immunosenescence is consist of a wide range of biomarkers alteration in the circulation system and shows its great potential in prediction of ArCSVD as a sensitive and accurate index available.

When ArCSVD initials and develops, multiple substances in circulation system experience drastic change. Inflammation-relative molecules, such as coding proteins and metabolites, have been the hotspot in this area. Several endothelial markers, involved in the activation of endothelial dysfunction and inflammation, were associated with the seriousness of CSVD, including neopterin, sICAM-1, and sVCAM-1 (112). Cytokines are another big family having great changes during the development of ArCSVD and divided into pro-inflammatory and anti-inflammatory subtypes based on their distinct functions. IL-6 and TNFα have raised great attention as their pro-inflammatory function in ArCSVD. The uplifting of serum IL-6 was usually observed in aged-related disease, and it was prone to be associated with the progression of ArCSVD (113). Other inflammatory factors, like TNF-α, were also increased in ArCSVD. It should be noted that the downward of anti-inflammatory cytokines also makes sense in ArCSVD as the newly discoveries in IL-10 (77, 114).

Obviously, patterns and related metabolites were also seriously affected by ArCSVD. The most significant changes are involving glycol metabolism, lipid metabolism, vitamin B and vitamin D metabolism pathways as what is tested in clinical practice. Glycosylated hemoglobin (HbA1c), low density lipoprotein (LDL), homocysteine (Hcy), 25-hydroxyvitamin D3 [25(OH)D3] stand for the representatives for the foregoing pathways and the first three substances significantly upregulates in ArCSVD while the last one as the sole protective factor decreases.

Immune cells also act a crucial role in ArCSVD and the detection of these cells helps physicians and specialists better predict this disease. The progressive remodeling of immune system displays as the shifts of cell subtypes, including NK cells, T cells and B cells subpopulations (115, 116). The diminishing of naïve T cell pool and increasing memory T cells may be the predictor of immunosenescence.

Today increasing evidence supports that non-coding RNAs have a great impact on development of ArCSVD. As the most well-known non-coding RNA, microRNAs have a more definitive function in a related area. MicroRNAs are a group of molecules that regulate the gene expression, mostly in a negative direction, and build a complicated interactive network in both pro- and anti-inflammatory pathways. Inflammation-associated microRNAs represent the conditions of immunosenescence such as MiR-126, MiR-146a, belonging to inflammatory pathway while also related to endothelial dysfunction, can be used for detection of ArCSVD (117, 118).

## The Potential Rejuvenation Strategies to Prevent CSVD During Aging

Immunosenescence accelerates and exacerbates CSVD while CSVD conversely promotes senescence of either CNS resident cells or immune cells. Thereafter, it is meaningful to prevent CSVD by postponing the process of immunosenescence. As inflammation is the crucial part of immunosenescence that damage the structure and function of nervous system in ArCSVD patients, most studies focus on anti-inflammation treatment.

Targeting key immune cells can be a potential approach to prevent or improve ArCSVD. Selectively inhibiting the activation or cytokines secretion of microglia and macrophages is a potential method to prevent the overactive neuroinflammation (119). Some studies found that regulating the gene expression and altering the phenotypes of microglia diminished the inflammation and promoted the recovery by drugs or chemotactic factors (120, 121). Besides, regaining the loss functions of immune cells can be another approach to prevent excessive inflammation. To clear the senescent cells, enhancement of phagocytosis in macrophages and chemotaxis in other immune cells and impeding the overproduction of pro-inflammatory cytokines are out of urgent necessity (122). In addition, the differentiation of Tregs, as well as its enhanced anti-inflammatory features, could be a prospective way to delay the progress of ArCSVD. Overall, preventing ArCSVD is still at the preclinical stage and not currently accepted in patients. Prior to clinical manifestations, further studies are required for better understanding the mechanism of interaction between anti-inflammation and ArCSVD treatment.

Actually, clinical practitioners reach a common consensus that lifestyle interventions may be a considerable and effective approach to alleviating CNS damage. The lifestyle intervention is well tolerated and it contains the management of sleep, motion and diet. The risk factors like hypertension, hyperlipidemia, hyperglycemia should be also controlled as they can disturb both the metabolism and inflammaging (82, 123, 124). Nutrition supplement, such as zinc, docosahexaenoic acid, active vitamin D, mecobalamin and folic acid, is also beneficial to the control of inflammation and immune response (125).

## Conclusion

CSVD is closely related to aging and little interest was shown in the contribution of immunity to CSVD according to previous studies. Nonetheless, the immunosenescence could be a prominent participator in the initiation and development of ArCSVD. So far, more studies should be carried out to further understand the association of immunosenescence and CSVD:(a) Whether the senescence of endothelia earlier than immunosenescence or not? (b) How immunosenescence disrupts blood-brain barrier step by step in this disease? (c) What should we do to uncover the specific targets connecting immunosenescence with CSVD under the rapid development of bioinformatic analysis and related technologies for sequencing? In all, the researchers take the responsibility to solve these questions and better transform the fundamental studies to the clinical practice.

## Author Contributions

BJ wrote the manuscript. MH and WC conducted the manuscript editing. BZ and ZL designed and critically revised the manuscript, supervised the project, and obtained fundings for this study. All authors contributed to the article and approved the submitted version.

## Funding

This work was supported by Guangzhou Science and Technology Program key project (202007030010), National Natural Science Foundation of China (81971110 to ZL) and the Guangdong Basic and Applied Basic Research Foundation (2020A1515010056).

## Conflict of Interest

The authors declare that the research was conducted in the absence of any commercial or financial relationships that could be construed as a potential conflict of interest.
